# Factors Associated with Significant Ocular Injury in Conservatively Treated Orbital Fractures

**DOI:** 10.1155/2014/412397

**Published:** 2014-12-16

**Authors:** Christopher J. Layton

**Affiliations:** Gallipoli Medical Research Institute, University of Queensland, Greenslopes Private Hospital, Greenslopes, QLD 4120, Australia

## Abstract

*Purpose.* To determine factors associated with the presence of significant ocular injury in subjects with orbital fractures. *Subjects.* A consecutive prospective cohort of 161 patients presenting to a general tertiary referral hospital with orbital fractures and undergoing initial conservative treatment was identified. Subjects were assessed at time of injury for the need for emergency surgery, and those initially treated conservatively were subsequently followed up by the Ophthalmology Department to assess for ocular injury requiring ophthalmic management at 1–7 days after injury. Associations between ocular injury and age, sex, visual acuity, presence of blowout fracture, extent of orbital involvement, and presence of distant facial fractures were assessed. *Results.* 142 male (average age of 32 [95% CI 30–35]) and 19 female (average age of 49 [95% CI 39–59]) subjects were identified. 17 subjects were diagnosed with significant ocular injury. Ocular injury was significantly associated with LogMAR VA worse than 0.2 (OR 49 [95% CI 11–217, *P* < 0.0001]), but no relationship was noted for age, sex, presence of blowout fracture, extent of fractures, or presence of distal facial fractures. LogMAR visual acuity worse than or equal to 0.2 had a 98% negative predictive value for ocular injury in the setting of orbital fractures. *Conclusions.* Demographic and nonophthalmic fracture characteristics were not useful predictors of ocular injury in orbital fractures. LogMAR visual acuity worse than or equal to 0.2 is a highly sensitive and useful guide of the need for ophthalmic referral in subjects with orbital fractures.

## 1. Introduction

Orbital fractures are a common traumatic condition more frequently occurring in young men and characterised by a wide range of clinical outcomes. Complications vary from bruising and temporary diplopia to traumatic optic neuropathy and heart block [1], and whilst some presentations require emergency orbital surgery with preoperative ophthalmic assessment, most fractures involving the orbital walls require only close observation or are observed before a decision to undertake delayed repair is made after several weeks [1]. In many jurisdictions, these assessments and treatments are carried out primarily by non-ophthalmologists.

The association between facial trauma and ocular damage seems logically unassailable and has been recognised since ancient times [2]; however, modern understanding of facial anatomy suggests the primary function of the hard anterior orbital rims and thin, easily broken orbital walls are to protect the eye in blunt trauma [1, 3]. This is possibly one reason why direct injury to the globe is relatively rare compared with the incidence of orbital fractures [4].

There are several studies which show that whilst severe ocular injuries are easily detected by the facial trauma surgeon or emergency physician, other subtle injuries are often missed [4, 5]. Therefore, one important clinical judgement for nonophthalmic physicians in caring for patients presenting with orbital fractures is to identify patients requiring specialist ophthalmological assessment. Fractures requiring emergency orbital surgery often undergo ophthalmological assessment as a safety precaution prior to surgical pressure being placed on the eye, however, the indications for ophthalmological referral in the large majority of orbital fractures which are initially treated conservatively are not clear.

Unfortunately, whilst available evidence to inform this judgement is extensive, it is often contradictory [6]. The incidence of ocular injuries of any severity in patients with orbital fractures has been variously reported to range from 2.7% [7] to 90% [8]. Recommended protocols for specialist ophthalmology screening have responded to this uncertainty by variably recommending referral of all orbital fractures [9], using formal risk scores [10], relying on the assessing physicians' clinical judgement (which in practice is often informed by bedside visual acuity measurements) [8] and using intraocular pressure as an indicator for referral [11]. Whilst studies investigating trauma can vary greatly based on local trauma patterns, other factors contribute to the apparent contradictions in the literature. These include only studying subjects who required surgery [12], using nonclinically relevant definitions of ocular trauma (e.g., inclusion of adnexal injuries or minor injuries not requiring ophthalmic intervention, like subconjunctival haemorrhage [8]), the retrospective nature of most studies [6], and the concentration on nonophthalmic (e.g., fracture classification or characteristics) over ophthalmic indicators in predicting an ophthalmic injury [13, 14]. To date only one prospectively planned study has been published which included subjects with orbital fractures who had not undergone surgery [8, 12].

This study aimed to prospectively assemble a sequential cohort of subjects to assess clinical indicators which may predict the need for ophthalmological treatment or follow-up in patients with orbital fractures undergoing initial conservative management. It hypothesises that the ophthalmic measurement of formally assessed habitually corrected visual acuity rather than demographic trends, injury type, or fracture characteristics is a sensitive and useful indicator for the presence of ocular injury in these patients. Further, it aims to determine if this measurement can form the basis of an efficient protocol for ophthalmology referral in these patients.

## 2. Methods

### 2.1. Subjects

A cohort of sequential presentations of patients with orbital fractures presenting over a 4-month period was identified at the Royal Brisbane and Women's Hospital, an inner city tertiary referral general hospital and the largest hospital in Australia. Institutional ethics review board approval was obtained and the study adhered to the Declaration of Helsinki. Subjects were assessed on presentation by facial trauma specialists for a positive oculocardiac reflex, CSF leakage, a penetrating mechanism of injury, extraocular muscle entrapment or injury, traumatic optic neuropathy, or other injuries to the face, cranium, or globe injury requiring emergency surgery. Affected patients were excluded from the study. 161 subjects with orbital fractures not requiring immediate surgery were subsequently examined at a routine ophthalmology review 1–7 days after injury in addition to their surveillance in the facial trauma clinic. Frequent communication between clinics took place to ensure a 100% referral rate was achieved. Subjects were excluded if they required presurgical assessment prior to emergency orbital surgery or if they were referred directly to the Ophthalmology Department with trauma that was primarily ophthalmic in nature, such as retrobulbar haemorrhage or ruptured globe. Subjects with bilateral orbital fractures had the side with the more extensive fractures, or if an ocular injury was present, the side with the ocular injury was included in the analysis. Patients later requiring deferred surgical repair after a period of observation were included in the analysis. Data were entered into a nonidentifiable database prepared by treating residents in the Ophthalmology Department. These examiners were masked regarding the study hypothesis. Age, sex, presence of inferior blowout fracture, number of involved orbital walls, presence of other facial fractures not involving the orbit, visual acuity, and type of ocular injury were recorded for all subjects.

### 2.2. Visual Acuity Measurement

Visual acuity was measured by residents in the Ophthalmology Department on ETDRS visual acuity charts at 4 meters, illuminated at 85 cd/m^2^ as recommended by the National Academy of Sciences Society Committee for Vision Testing Standards. Corrected visual acuity was recorded in LogMAR notation with the subject's habitual visual correction. For the purposes of analysis only, vision better than 0.2 was classified as “good visual acuity,” whilst vision equal to or worse than 0.2 was classified as “poor visual acuity.”

### 2.3. Definition of Significant Ocular Injury

Significant ocular injury was defined as any injury requiring ophthalmic treatment or ongoing specialist ophthalmology follow-up. Diplopia, soft tissue swelling, conjunctival chemosis, isolated subconjunctival haemorrhage, and mild, nonmacular involving commotio retinae were specifically excluded. Injuries identified in either the eye associated with the fracture or the uninvolved eye were analysed as an injury associated with the orbital fracture.

### 2.4. Statistical Analysis

Results were analysed with SPSS version 22 in conjunction with medical statistics service StatAid. The impact of each presenting factor on the presence of significant ocular injury was assessed by binary logistic regression with Hosmer-Lemeshow testing. Significant associations were further assessed by the Fisher exact test. Differences in means were assessed with the Mann-Whitney test. All values are expressed with 95% confidence intervals.

## 3. Results

**Figure 1:**
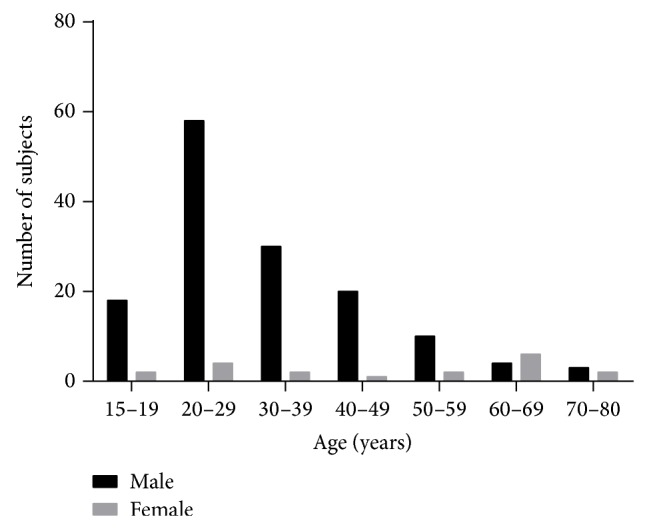
Subject demographics. Orbital fractures were more frequent in men and peaked in the third decade of life. 142 subjects were male with an average age of 32 [95% CI 30−35] years. There were 19 female subjects with an average age of 49 [95% CI 39–59] years. The difference in age of presentation was significant (*P* < 0.001).

Clinical data collection from all 161 subjects was complete. 142 subjects were male with an average age of 32 [95% CI 30−35] years, and 19 subjects were female with an average age of 49 [95% CI 39–59] years (Figure 1). This difference in the age of presentation between the sexes was statistically significant (*P* < 0.001). 103 subjects had blowout fractures and 24 had fractures of facial bones distant to the orbit. 16 patients had orbital fractures involving both orbits. Average habitually corrected LogMAR visual acuity for the cohort was 0.07 [95% CI 0.03–0.1]. Characteristics of the study group are shown in Table 1.

17 of the 161 subjects were found to have significant ocular pathology as shown in Table 2. Two further subjects had unrelated ophthalmic pathology diagnosed during assessment (Marfan's syndrome and amblyopia). These diagnoses were not included in the group with ocular injuries. Mean visual acuity in the group diagnosed with significant ocular injuries was 0.44 [95% CI 0.20–0.68] and in those without was 0.02 [95% CI 0.00–0.05]. This difference was statistically significant (*P* < 0.001). 89% of all subjects had subconjunctival haemorrhage, 7 subjects had minor peripheral commotio retinae, and no subjects had corneal abrasions.

**Figure 2:**
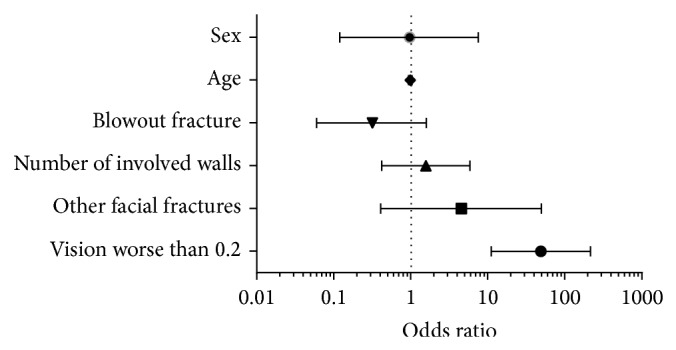
Fisher plot of possible predictors of ocular injury in orbital fractures not requiring emergency surgery. Age, sex, type of fracture, extent of fractures within the orbit, and presence of distal facial fractures were not statistically associated with the presence of significant ocular injury. Habitually corrected visual acuity was significantly associated with the presence of significant ocular injury (OR 49 [95% CI 11–217, *P* < 0.0001]).

Subjects were subsequently categorised as either having visual acuity better than 0.2 or worse than or equal to 0.2. The results of the binary logistic regression are shown in Figure 2. A statistically significant odds ratio was associated with the finding of significant ocular injury in subjects with visual acuity of worse than or equal to 0.2 (OR 49 [95% CI 11–217, *P* < 0.0001]). There was no significant association between the presence of an ocular injury and sex (OR 0.96 [95% CI 0.1–7.5]), age (OR 0.99 [95% CI 0.94–1.04]), number of involved orbital walls (OR 1.6 [95% CI 0.4–5.9]), or presence of blowout fracture (OR 0.3 [95% CI 0–1.6]). Only one subject with distant facial fractures was diagnosed with a significant ocular injury, but the presence of ocular injury in subjects with distant facial fractures was not significant (*P* = 0.22). 3 subjects with an ocular injury had visual acuity better than 0.2. One of these subjects had a retinal tear in the uninvolved eye, whilst the other two were diagnosed with traumatic iritis.

**Figure 3:**
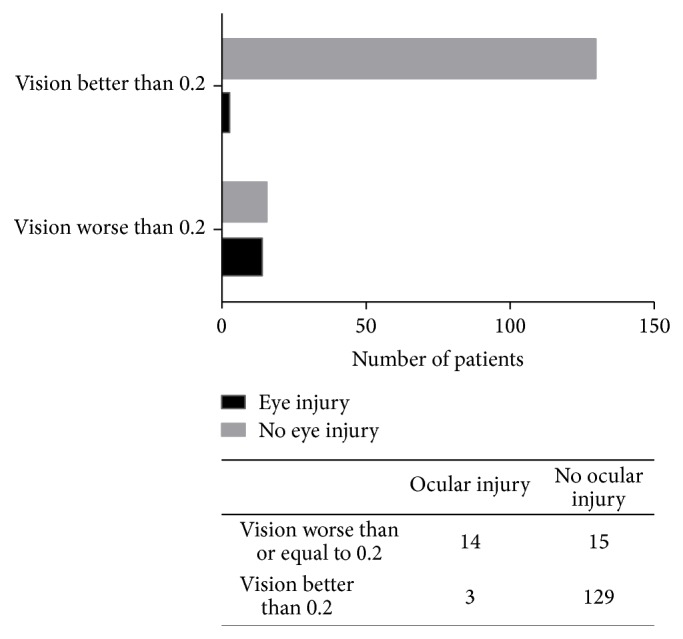
14 of 17 subjects with coexistent orbital fractures and ocular injuries had habitual visual acuities worse than or equal to 0.2 LogMAR. Habitually corrected visual acuity worse than or equal to 0.2 was 82% sensitive and 90% specific for ocular injury, with a negative predictive value of 98%.

Visual acuity worse than or equal to 0.2 was 82% sensitive and 90% specific for the presence of ocular injury. The negative predictive value (i.e., probability of visual acuity better than 0.2 indicating no ocular injury) was 98% [95% CI 94%–99%] (Figure 3).

## 4. Discussion

This study is the first prospective study to investigate ocular injuries only in those orbital fractures that do not require immediate surgery and undergo initial conservative management and observation. This is an important group in clinical practice because patients with these fractures form the majority of orbital fracture presentations, are assessed in controlled clinic situations where accurate measurements to inform the referral decision are possible, and because other orbital fractures requiring emergency repair require ophthalmic evaluation to prevent significant vision loss from exacerbation of ocular injury during orbital surgery [12]. Therefore, the inclusion criteria in this study capture the majority of patients in whom the decision to undertake specialist ophthalmic assessment is unclear.

The results presented here show that the presence of visual acuity worse than or equal to 0.2 is a highly sensitive and specific predictor for ocular injury in this cohort and that none of the nonophthalmic indicators tested were found to be helpful in predicting the presence of ocular trauma in these subjects.

The overall incidence of ocular injury requiring ophthalmic treatment or follow-up in this cohort was 10.6%, significantly lower than some studies which have reported incidences up to 90% [8]. Possible contributions to the higher incidences in these other studies are the inclusion of nonsignificant ophthalmic injuries such as subconjunctival haemorrhage [8] and inclusion of subjects already requiring ophthalmic assessment prior to emergency orbital surgery or for a primary diagnosis of ocular trauma [12]. One retrospective study excluded subconjunctival haemorrhage and reported an incidence of ocular injury of 15.6%, finding the most commonly associated ocular injury in surgically treated orbital fractures to be corneal abrasion [15]. In the protocol of the present study ophthalmic assessment was usually delayed by several days due to the routine nature of the injuries and any abrasions present on diagnosis would have healed naturally before ophthalmic examination.

Two previous studies allow subgroup analysis using similar definitions of ocular injury to this study and therefore allow direct comparison to the results presented. In the first, the only other large prospective trial including conservatively managed subjects, subgroup analysis shows that 11.6% of subjects suffered from significant ocular injury. In the second, a large retrospective review, the reported incidence of ocular injury was 10.3% [16]. Both figures agree closely with the 10.6% incidence reported here.

In this cohort there was no relationship between the type or extent of orbital fracture and ocular trauma. Previous studies report contradictory results regarding the relationship between orbital fracture type and ocular injury. Traditionally, blowout fractures have been considered more likely to be associated with ocular injury due to the hydraulic (or globe to wall) theory of blowout aetiology [17], and this has been supported by retrospective data in 126 subjects reported by Mellema et al. [18] and experimental work in monkeys [19]. However, other investigators have found that poor visual acuity and blindness are more likely to be due to zygomatic fractures [6, 13]. The finding that ocular injury is not related to the type or extent of orbital fractures in this cohort of subjects is supported by a retrospective analysis of 4082 facial fractures, in which no relationship between ocular injury and type of fracture was found [16]. These findings could be argued to support a buckling mechanism as the cause of a majority of blowout fractures in the present study group.

In this study ocular injury requiring ophthalmic treatment or follow-up was associated with approximately 1 in 10 orbital fractures undergoing conservative management in the initial stages. Therefore, in many settings, routine referral of all orbital fractures may not be practical or economical, however, nonreferral of a serious occult ocular injury could have serious consequences. As such, a high negative predictive value is of primary importance in any referral protocol.

In this study, a predetermined habitually corrected visual acuity threshold of less than or equal to 0.2 resulted in a negative predictive value for ocular injury of 98% and had the potential to reduce the number of specialist ophthalmic assessments from 161 to 29 during the study period. Some comparison of this finding to previous studies is possible, with subgroup analysis of the prospective study by Al-Qurainy et al. [10]; showing a Snellen visual acuity threshold for referral of 6/12 alone gives a sensitivity for ocular injury of 80%, essentially equivalent to the 82% reported here. This previous study investigated both emergency and conservatively managed orbital fractures from the west of Scotland and found that the negative predictive value for this cohort could be improved to a maximum of 87% by including a small weighting for a complex scoring system involving fracture type, blowout fracture, diplopia, and amnesia. The higher negative predictive value in the present study is possibly due to the exclusion of fractures requiring immediate treatment in this cohort and a more formal approach to visual acuity testing. Both this study and that reported by Al-Qurainy found that most patients with normal visual acuity who were diagnosed with ocular injury suffered from anterior uveal damage [10], which is particularly difficult to diagnose for the nonophthalmologist.

This study was carried out in a single large general tertiary referral centre, and the characteristics of trauma and local management protocols can be expected to naturally vary between regions and between facility types within a region. Despite this, the reported incidence of significant ocular injury, the sensitivity of decreased visual acuity as an indicator of this injury, and the lack of association of ocular injury with type of fracture found in this cohort are aligned with other reports, once subgroup analyses of previous retrospective studies and the only other prospective study are considered. Further local studies and reviews are nonetheless necessary to confirm the value of visual acuity as a predictor of ocular injury in orbital fractures in specific centres.

In centres where routine referral of all orbital fractures is impractical or uneconomical, the results from this cohort suggest that the routine accurate measurement of habitually corrected visual acuity in patients with orbital fractures has a very high negative predictive value in informing the need for ophthalmic assessment. This study excluded patients undergoing emergency orbital surgery in order to isolate that patient group in whom the decision to refer for specialist ophthalmology opinion is unclear. Therefore the results are not directly applicable to presentations where emergency surgery is required or where the criteria for emergency surgery used in this study are met, and particular caution should be exercised in considering these limitations in clinical settings where ophthalmic consultation is difficult to arrange or unavailable. It should also be noted that, although visual acuity measurement is able to be performed routinely by non-ophthalmology trained physicians and assistants, accurate acuity measurement does require some minor training, modern visual acuity charts, and attention to testing conditions. As this study focused on patients already under observation for their fractures in a facial trauma clinic, the difficulties of measuring visual acuity at the bedside can be avoided in this group and therefore accurate measurement can usefully inform referral decisions.

## Figures and Tables

**Table 1:** Group characteristics.

Age	
Male	32 [95% CI 30–35] years
Female	49 [95% CI 39–59] years
Sex	
Male	142
Female	19
Blowout fracture	
Yes	103
No	58
Involved orbital walls	
1	70
2	87
3	4
Distant facial bone fractures	
Yes	25
No	136
Best corrected visual acuity	0.07 [95% CI 0.03–0.1]
Better than 0.2 Log⁡MAR	132
Worse than or equal to 0.2 Log⁡MAR	29
Ocular injury	
Yes	17
No	144

**Table 2:** Observed injuries.

Injury	Number of cases
Retinal tear	2
Pigment epithelial detachment	1
Commotio retinae involving macula	5
Hyphema	4
Traumatic iritis	3
Traumatic optic neuropathy	1
Iris tear	1
